# Deep learning-based magnetic resonance image segmentation technique for application to glioma

**DOI:** 10.3389/fmed.2023.1172767

**Published:** 2023-11-20

**Authors:** Bing Wan, Bingbing Hu, Ming Zhao, Kang Li, Xu Ye

**Affiliations:** ^1^Department of Radiology, China Three Gorges University, Affiliated Renhe Hospital, Yichang, Hubei; ^2^School of Computer Science, Yangtze University, Jingzhou, China; ^3^Department of Radiology, Chongqing People’s Hospital, Chongqing, China; ^4^Electronics and Information School, Yangtze University, Jingzhou, China

**Keywords:** deep learning, brain tumor segmentation, magnetic resonance imaging, loss function, medical image

## Abstract

**Introduction:**

Brain glioma segmentation is a critical task for medical diagnosis, monitoring, and treatment planning.

**Discussion:**

Although deep learning-based fully convolutional neural networks have shown promising results in this field, their unstable segmentation quality remains a major concern. Moreover, they do not consider the unique genomic and basic data of brain glioma patients, which may lead to inaccurate diagnosis and treatment planning.

**Methods:**

This study proposes a new model that overcomes this problem by improving the overall architecture and incorporating an innovative loss function. First, we employed DeepLabv3+ as the overall architecture of the model and RegNet as the image encoder. We designed an attribute encoder module to incorporate the patient’s genomic and basic data and the image depth information into a 2D convolutional neural network, which was combined with the image encoder and atrous spatial pyramid pooling module to form the encoder module for addressing the multimodal fusion problem. In addition, the cross-entropy loss and Dice loss are implemented with linear weighting to solve the problem of sample imbalance. An innovative loss function is proposed to suppress specific size regions, thereby preventing the occurrence of segmentation errors of noise-like regions; hence, higher-stability segmentation results are obtained. Experiments were conducted on the Lower-Grade Glioma Segmentation Dataset, a widely used benchmark dataset for brain tumor segmentation.

**Results:**

The proposed method achieved a Dice score of 94.36 and an intersection over union score of 91.83, thus outperforming other popular models.

## 1 Introduction

Gliomas are the most common primary cranial tumors, and they arise from cancerous glial cells in the brain and spinal cord. In accordance with their malignancy level, gliomas are classified as low or high grade. For most glioma patients, early detection, complete surgical excision, and postoperative treatment with radiotherapy and chemotherapy yield good results (1). Moreover, recent studies in this field have identified an association between the tumor shape features extracted from magnetic resonance imaging (MRI) images and their genomic subtypes (2, 3). However, the first step in tumor feature extraction is manual segmentation of MRI images, which is a costly and time-consuming process. Moreover, the resulting annotations may have high inter-rater variance (4). With the rapid development of neural network technology, applications of convolutional neural networks (CNNs) with robust feature extraction ability in the field of medical imaging have revealed distinct advantages. As a result, computer vision technology is now widely used in medical image analysis.

Image segmentation is a highly important step in medical image analysis. Early image segmentation methods mainly employed threshold- (5), region- (6), edge detection- (7), and cluster-based image segmentation algorithms. The advantages of these algorithms were their simple calculations and high efficiencies. However, single image features were obtained, and spatial features were neglected; thus, these algorithms were sensitive to noise and had low robustness. In recent years, deep learning-based medical image methods have become mainstream because of their superior results. In 2015, (8) pioneered the fully convolutional network, which replaced the fully connected layer in the traditional CNN with a convolutional layer. Using this method, image segmentation maps of any size could be generated. Thus, similar structures were adopted for almost all subsequent semantic segmentation fields. In another notable development, (9) proposed the U-Net network, which is characterized by a cross-layer connection structure; this structure enables simultaneous integration of low- and high-level semantic information in the feature map, yielding improved segmentation results. Moreover, the DeepLab (10) series of semantic segmentation networks was developed by Google. The DeepLab model uses a variety of expansion convolutions with different coefficients to extract image information from different receptive fields; its performance has greatly improved over time.

Current medical image segmentation methods mainly rely on fully convolutional neural networks (11) with U-shaped structures (9, 12, 13). Although these techniques have performed general medical image segmentation tasks with great success, specific challenges remain. Most segmentation models have similar feature extraction processes: the input image is continuously reduced in width and height while its depth is increased to obtain its higher-level features. Then, in the feature recovery phase, the features are recovered through continuous deconvolution or interpolation; hence, a prediction with the same width and height as the input image is obtained. During the feature recovery phase, a large number of hyperparameters, such as the convolution kernel size, number, step, and activation function, are manually set. Therefore, there is considerable reliance on researcher experience and prior knowledge, and avoiding redundant structures is difficult. As a result of the inherent limitations of human knowledge, escaping the original paradigm to design an optimal model is a challenge (14).

In most cases, a neural architecture search (NAS) can yield an effective model (15). This type of model structure has the advantages of a strong automatic acquisition generalization ability and low hardware requirements; thus, the optimal network-structure parameter configuration can be found quickly and accurately with the appropriate search strategy. The RegNet design-space search technique (16) is similar to the NAS method but can search for simpler, easier-to-understand, and easier-to-quantify design spaces. Since its introduction, refinements of the RegNet design space (17, 18) have focused on the optimization of the sample size and the model scaling strategy. The RegNet generic model employs a similar feature extraction process to other segmentation models, with the same constant reduction in width and height accompanied by an increase in depth. In contrast, RegNet combines the advantages of NAS and manual design so that a model architecture with better network-structure parameters, interpretability, performance, and effectiveness is devised.

To devise a medical image segmentation model, a model decoder is required. DeepLabv3+ (19) requires only fusion of the bottom- and top-level features and is significantly less redundant than conventional cross-layer connected segmentation algorithms. Therefore, a combination of RegNet and DeepLabv3+ can be expected to yield a less redundant structure and superior network-structure parameters than manually designed networks.

The loss function must also be considered when designing an effective medical image segmentation method. Many semantic-based segmentation loss functions focus on pixel-level classification errors but ignore pixel-level structural information such as the cross-entropy loss. In addition, the segmentation results obtained with conventional loss functions often contain noisy segmentation regions, implying segmentation errors. Although these regions are small, they affect the final segmentation quality; therefore, their presence is an additional concern. Thus, appropriate loss functions are required to overcome these challenges. Notably, the loss function adapted from the Dice coefficient, which is called the Dice loss (20), can solve the problem of sample imbalance.

In the context of glioma, the Lower-Grade Glioma (LGG) Segmentation Dataset (2) contains MRI images and text data. The MRI image shape features are closely related to the LGG genomic subtype and, thus, the patient prognosis. These shape features also contain hidden information on the slice order. All these data can provide feature information for segmentation of the model (3). Therefore, an attribute information encoder that can fuse image, text, and slice-order features by fully exploiting the existing multimodal data information should be designed.

In this study, an MRI image segmentation model that combines image, text, and slice sequence data is designed, in which an advanced NAS model is employed as an encoder for improved image feature extraction. The Dice loss is used to solve the sample imbalance problem, and the use of the outlier loss to remove noise from the segmentation results is proposed.

The remainder of this paper is organized as follows. Section 2 summarizes related work, and Section 3 presents the main methods and details of the proposed segmentation model. Section 4 reports the implementation details and experimental results. Finally, Section 5 presents the limitations and conclusions of the study.

## 2 Related work

### 2.1 MRI segmentation of brain tumors

In recent years, many deep learning-based methods, such as the widely used CNNs, have been proposed for brain tumor MRI segmentation. Based on the processing dimension, deep learning-based brain tumor MRI segmentation methods can be divided into two categories: methods based on 2D and 3D CNNs. Methods based on 2D CNNs primarily use the traditional sliding window method to independently predict brain tumors for each image slice (21); this limits the segmentation accuracy as the depth information of the image is not used. By contrast, methods based on 3D CNNs can effectively use the depth information of the image and improve the segmentation accuracy. However, they suffer from huge computational cost, making their application to practice difficult. This study encodes the depth information as a sequence and integrates it into a 2D CNN, which combines the advantages of speed and accuracy.

### 2.2 Encoder–decoder architecture

The encoder–decoder structure (9, 22–25) is a very popular engineering architecture for the performance of many computer vision tasks, such as object detection (26, 27) and semantic segmentation (28–30), and helps to functionally decouple the backbone model from the generative model. Recently, RefineNet (26) and related approaches (25, 31, 32) demonstrated the effectiveness of models based on encoder–decoder architecture by reaching several semantic segmentation benchmarks (19). In this structure, the encoder module usually serves as a logical representation of the backbone network, acquiring features from the input and outputting them as fixed shapes. In this process, the spatial dimensionality of the feature mapping gradually decreases, enabling the capturing of a wider information range within a deeper encoder output. The decoder serves as a logical representation of the upsampling network, gradually obtaining clear object boundaries. Based on this architecture, in the model proposed in the present study, RegNet is used as an image encoder for image feature extraction, a multilayer perceptron (MLP) is used as an attribute encoder for attribute feature extraction, an atrous spatial pyramid pooling (ASPP) module (19) is used as a further feature extractor and integrator, and DeepLabv3+ is employed as a decoder to generate segmentation results.

### 2.3 RegNet

The NAS process automatically designs neural network structures using neural networks. This contrasts with the structural design of deep CNNs, which requires considerable expertise and time. However, NAS has significant limitations in this search space, along with insufficient interpretability (17). Radosavovic et al. of Facebook AI Research combined the advantages of manual design and a NAS to derive a RegNet with a low-dimensional design space consisting of simple, regular networks. The RegNet model obtained in this manner had superior interpretability, performance, and results to manually designed networks. Therefore, the RegNet model is employed as an image encoder for image feature extraction in this work.

### 2.4 DeepLabv3+

As mentioned above, DeepLab refers to a series of semantic segmentation networks proposed by Google. Among them, DeepLabv3+ extends DeepLabv3 (33), with the main improvement being abstraction of the original DeepLabv3 single-model structure to an encoder–decoder structure. In addition, the ASPP module is retained; this module extracts image information from different sensory fields through several parallel atrous convolutions with different rates, before fusing them into deep image features through channels. In the decoder component, the low and high-level features are fused to generate the final segmentation result. In the present study, the decoder component of DeepLabv3+ is used for image feature recovery and to generate final segmentation results.

### 2.5 Loss function in image segmentation

The loss function most commonly applied to image segmentation is the distribution based cross-entropy loss. This loss function treats the segmentation problem as a pixel-by-pixel classification problem, calculates the cross-entropy loss for each pixel individually, and averages or sums over all pixels. The binary cross-entropy (BCE) loss function is often used in cases involving positive and negative samples only. However, this loss function neglects the differences in proportions between samples of different categories. Thus, in training, the model is biased toward the category with more samples and sample imbalance occurs. This imbalance causes training inefficiency and difficulty in learning useful learning signals, thereby reducing the network effectiveness (34). Notably, The LGG Segmentation Dataset also suffers from a sample imbalance problem.

The Dice coefficient is used to calculate the similarity between two images, and the loss function adapted from this coefficient is called the Dice loss. Application of the Dice loss can solve the sample imbalance problem; however, this loss is often combined with other loss functions, with the disadvantage of training instability.

In this study, the above loss functions are weighted to combine the advantages of each, and the outlier loss, a new loss function for suppressing specific size regions, is proposed. Hence, the final Dice similarity coefficient (DSC) and the segmentation quality are improved.

## 3 Novel deep learning model based on DeepLabv3+

Section “2 Related work” explained the reasoning behind the selection of the overall architecture of the proposed deep neural network model and briefly introduced the functions of the main modules. These modules constituted the basic units of the designed model, with custom modifications being implemented to accommodate the input shape requirements. This section presents the overall model architecture and the main encoder and decoder functions and analyzes the principles and usage of the main loss functions.

### 3.1 Architecture overview

**FIGURE 1:**
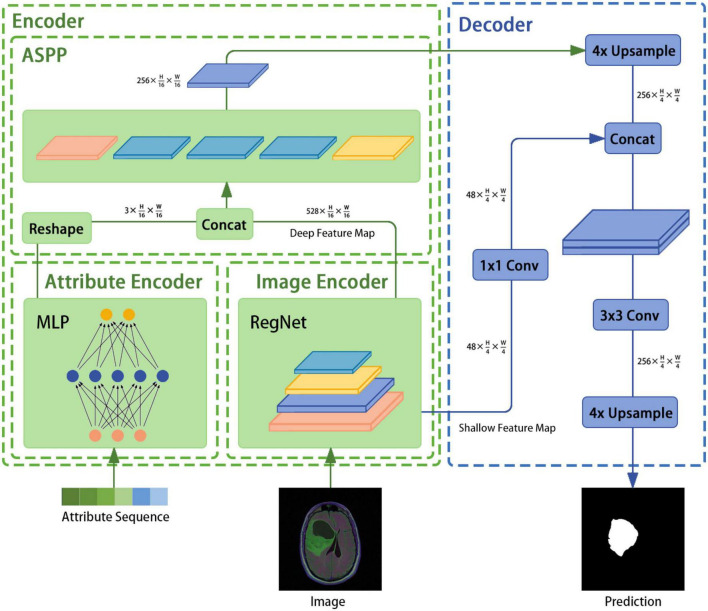
Overview of the image segmentation model based on encoder–decoder architecture.

The architecture of the image segmentation model developed in this study is shown in Figure 1 and consists of an attribute encoder, an image encoder, an ASPP module, and a decoder. The attribute encoder comprises a simple MLP, and the image encoder comprises a RegNet model. The ASPP module extracts semantic information from different sensory fields and plays a fusion role, and the decoder is taken from DeepLabv3+.

### 3.2 Encoder

The encoder mainly consists of the image encoder RegNet module, the attribute encoder, and the ASPP module. An image with a known size of H × W × 3 is input to the image encoder and the following output is generated: $\left\{48\,\text{×}\,\frac{H}{4}\,\text{×}\,\frac{W}{4},528\,\text{×}\,\frac{H}{16}\,\text{×}\,\frac{W}{16}\right\}$.

#### 3.2.1 Image encoder

Radosavovic et al. (17) of Facebook AI Research proposed model distribution in the design space to estimate the design-space quality. They used the empirical distribution function as an evaluation metric:


(1) $$F\left(e\right)=\frac{1}{n}\sum_{i=1}^{n}1\left[e_{i}<e\right],$$

where 1 is the indicator function, *n* is the number of given models, *e_i_* is the set of model errors, and F(*e*) gives the fraction of models with error less than *e*.

Nearly every neural network design can be abstracted into three modules: the input layer stem, the backbone layer body, and the output layer head. In this study, an initial design space, AnyNetX, was defined according to this architecture and optimized in the body module only, which gradually develops the initial design space to the final RegNet design space. As a result, the number of samples in the design space was reduced from 1.8×10^18^ to 3×10^8^. Hence, a higher-quality design space was obtained.

**FIGURE 2:**
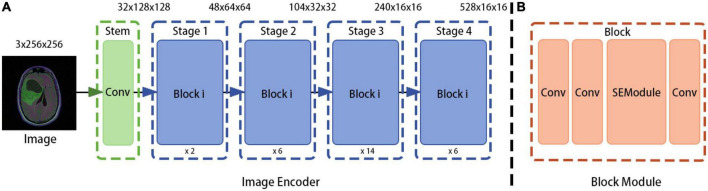
RegNet (regnetz_040) model architecture. **(A)** The image encoder consists of a stem and a body. **(B)** The network body is composed of a series of blocks, which contain convolution modules and Squeeze-and-Excitation (SE) channel attention modules, aimed at evaluating the importance of channel dimensions and more efficiently extracting features (9).

In the proposed method, the model obtained from the RegNet design space is used as the image encoder. Again, the network is mainly composed of a stem, body, and head. The stem is a general convolution layer (including BN and SiLU by default), the convolution kernel size is 3 × 3, the stride is 2, and the number of convolution kernels is 32. The body is composed of four stacked stages, as shown in Figure 2. After each stage, the input matrix height and width are halved. Each stage is composed of a series of block stacks. In the first block, group and general convolutions with a stride of 2 are performed; for the remaining blocks, the convolution stride is 1. The head is a common classifier in a classification network, which consists of a global average pooling layer and a fully connected layer.

To design the proposed model, the encoder input structure was fine-tuned; for example, the head structure was removed and the results of the first and last stages were saved and outputted. In addition, the image encoder was designed to receive image input with dimensions of 3 × H × W and to output $\left\{48\,\text{×}\,\frac{H}{4}\,\text{×}\,\frac{W}{4},528\,\text{×}\,\frac{H}{16}\,\text{×}\,\frac{W}{16}\right\}$, where $48\,\text{×}\,\frac{H}{4}\,\text{×}\,\frac{W}{4}$ is output directly as the bottom feature and $528\,\text{×}\,\frac{H}{16}\,\text{×}\,\frac{W}{16}$ is sent to the ASPP module as a high-level feature (after fusion with the attribute encoder output).

#### 3.2.2 Attribute encoder

In addition to MRI image data and genome cluster table data for patient brains, the LGG dataset includes location data for the image slices, which may constitute valid information for the final segmentation results. For example, there is a higher probability of obtaining segmentation results for large areas when the slice images are obtained at intermediate locations compared to the probability when these images are taken at edge locations. This tendency suggests that the segmentation results may be related to the slice location data. A previous study demonstrated that the shape features in MRI images are strongly associated with genomic subtypes and patient outcomes in LGG; thus, it was also necessary to incorporate patient genomic cluster data in the proposed model (3).

An attribute encoder extracts text and position information and mainly consists of an MLP. The specific operations are as follows: (1) genome cluster encoding through one-hot encoding, (2) implementation of the concatenation operation on the above encoding result and the position information to generate a sequence of length N, (3) inputting this sequence into the attribute encoder, and (4) outputting a feature with dimensions $3\,\text{×}\,\frac{H}{16}\,\text{×}\,\frac{W}{16}$ after the reshaping operation.

#### 3.2.3 ASPP module

The ASPP structure, which was first proposed for DeepLabv2 (33) and then improved in DeepLabv3, uses several parallel atrous convolutions with different rates to fuse multiscale information. Hence, semantic information is combined with different perceptual fields, which is crucial for segmentation accuracy. First, the ASPP receives feature maps of size $528\,\text{×}\,\frac{H}{16}\,\text{×}\,\frac{W}{16}$ and $3\,\text{×}\,\frac{H}{16}\,\text{×}\,\frac{W}{16}$ from the image and text encoders, respectively. Second, the dual-modal fusion work of the feature maps is completed through the concatenation operation and the result is used as the feature input. Third, the result is subjected to one 1 × 1 convolution, three 3 × 3 convolutions with rates of (6, 12, 18), and a pooling layer. The concatenation operation is then performed to adjust the number of channels through a 1 × 1 convolution. Finally, the encoder feature is output.

### 3.3 Decoder

In this study, the decoder component of DeepLabv3+ is employed directly. In DeepLabv3, direct upsampling of feature maps is performed eight times. However, a decoder module is introduced to upsample and fuse the underlying and high-level features; this provides richer semantic information and a better edge segmentation effect. First, high-level features of size $256\,\text{×}\,\frac{H}{16}\,\text{×}\,\frac{W}{16}$ are restored to feature maps of size $256\,\text{×}\,\frac{H}{4}\,\text{×}\,\frac{W}{4}$ through applying bilinear interpolation four times. Then, the channel number of the low-level features of size $48\,\text{×}\,\frac{H}{4}\,\text{×}\,\frac{W}{4}$ is adjusted through a 1×1 convolution. After the two feature types are processed by concatenation, a 3 × 3 convolution is used to further fuse them. Finally, a bilinear interpolation is applied four times to obtain a segmentation prediction map of the same size as the original image.

### 3.4 Loss function

#### 3.4.1 Cross-entropy loss function

Cross-entropy is defined as a measure of the difference between two probability distributions for a given random variable or set of events. This measure is widely used for classification tasks and works well for segmentation tasks as they can be viewed as pixel-level classification tasks. The cross-entropy loss function can be applied to most semantic segmentation scenarios. However, when the numbers of foreground and background pixels differ, the training is biased toward the category with more samples; this causes sample imbalance and, hence, inefficient training.

The LGG dataset has only foreground and background categories; therefore, the BCE loss can be used:


(2) $$\begin{array}{c} L_{B\,C\,E}\,\left(X,Y\right)=-\left(Y\,\text{⋅}\,l\,o\,g\,X+\left(1-Y\right)\,\text{⋅}\,l\,o\,g\,\left(1-X\right)\right) \end{array}$$

#### 3.4.2 Dice loss function

The Dice coefficient is widely used in the field of computer vision as a metric to calculate the similarity between two images. In 2016, this coefficient was adapted to a loss function, which is called the Dice loss:


(3) $$\begin{array}{c} L_{\text{Dice}}\,\left(X,Y\right)=1-\frac{2\,\left|X\cap Y\right|+1}{\left|X\right|+\left|Y\right|+1}, \end{array}$$

where |*XY*| denotes the intersection between *X* and *Y*, and |*X*|and |*Y*|denote the number of elements of *X* and *Y*, respectively. Note that the coefficient in the numerator is 2 because the common elements of *X* and *Y* are double counted in the denominator.

#### 3.4.3 Outlier-region loss function

**FIGURE 3:**
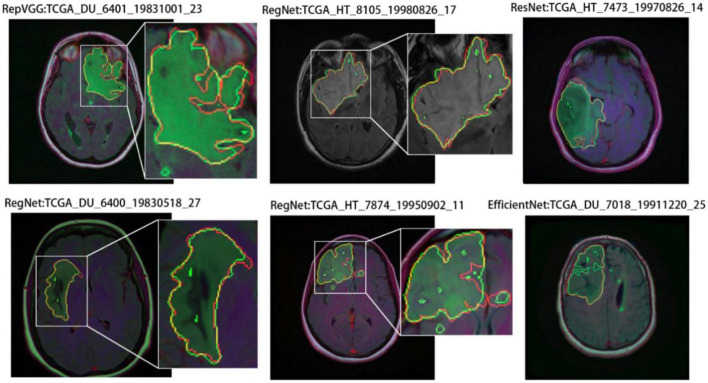
Segmentation results for regions with noise in parts. The red and green borders indicate the true and predicted labels, respectively.

In this study, various models were trained on the LGG dataset (see section “4.2.2 Ablation analysis of multimodal data”). Their segmentation results included noisy segmentation regions, as shown in Figure 3. As noted previously, such noisy regions often indicate segmentation errors. As confirmed by a segmentation size analysis for the dataset (see section “4.1.3 Implementation details”), the noisy segmentation areas were small. However, they affected the final segmentation quality; therefore, they required attention.

**FIGURE 4:**
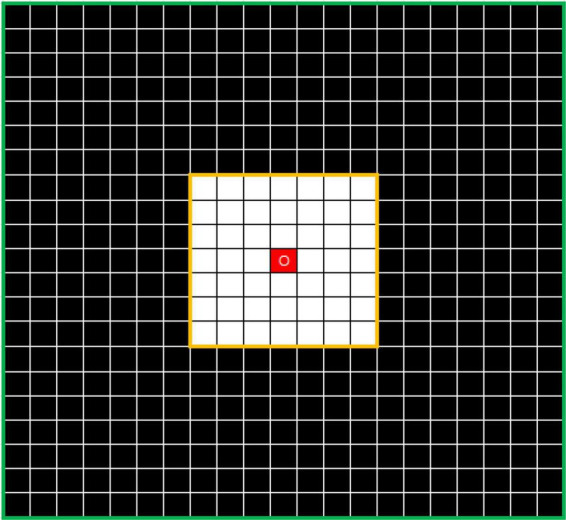
Example showing *N*_*7*_ and *N*_*21*_ neighborhoods centered on O. The value of *DensityWhite*(*O*) is the ratio of the number of white dots to the number of black dots.

To solve this problem, the outlier loss, which is a density-based loss function mainly used to suppress generation of a specifically sized region, was proposed for outlier-region detection. The underlying concept of the outlier loss function is that the density of the area in which a point is located is taken as a loss indicator. As an example, the characteristics of white noise on a black background are shown in Figure 4.

In this approach, the *k* × *k* neighborhood *N_k_* is divided, with point O as its center. The numbers of white and black points in *N_k_* are defined as *CountWhite*(*N*_*k*_) and *CountBlack*(*N*_*k*_), respectively. Thus, the number of interval points can be written as


(4) $$\begin{array}{c} C\,o\,u\,n\,t\,G\,a\,p\,W\,h\,i\,t\,e\,\left(X\right)=C\,o\,u\,n\,t\,W\,h\,i\,t\,e\,\left(N_{j}\right)-C\,o\,u\,n\,t\,W\,h\,i\,t\,e\,\left(N_{i}\right), \end{array}$$

(5) $$\begin{array}{c} C\,o\,u\,n\,t\,G\,a\,p\,B\,l\,a\,c\,k\,\left(X\right)=C\,o\,u\,n\,t\,B\,l\,a\,c\,k\,\left(N_{j}\right)-C\,o\,u\,n\,t\,B\,l\,a\,c\,k\,\left(N_{i}\right), \end{array}$$

where the input *X* is an *n* × *n* prediction mask matrix, the sigmoid function is used for processing, *X* ∈ (0,1), the output has the same shape as the input, *i* and *j* are hyperparameters, and *j* > *i*. The white and black point densities can be defined as


(6) $$\begin{array}{c} D\,e\,n\,s\,i\,t\,y\,W\,h\,i\,t\,e\,\left(X\right)=\frac{C\,o\,u\,n\,t\,W\,h\,i\,t\,e\,\left(N_{i}\right)+s\,m\,o\,o\,t\,h}{C\,o\,u\,n\,t\,G\,a\,p\,B\,l\,a\,c\,k\,\left(X\right)+s\,m\,o\,o\,t\,h}, \end{array}$$

(7) $$\begin{array}{c} D\,e\,n\,s\,i\,t\,y\,B\,l\,a\,c\,k\,\left(X\right)=\frac{C\,o\,u\,n\,t\,B\,l\,a\,c\,k\,\left(N_{i}\right)+s\,m\,o\,o\,t\,h}{C\,o\,u\,n\,t\,G\,a\,p\,W\,h\,i\,t\,e\,\left(X\right)+s\,m\,o\,o\,t\,h}, \end{array}$$

respectively, where smooth is the smoothing coefficient used to avoid division by 0.

**FIGURE 5:**
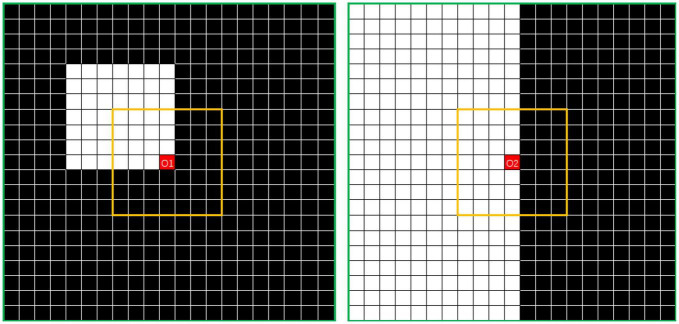
Distributions of noise-area corner (left) and segmented-object border area (right).

The density characteristics of the corner area of the noise and those of the edge area of the segmented object should be distinguished. In Figure 5, where *i* is 7 and *j* is 21, the points O1 in the left image and O2 in the right image represent the pixel at the corner of the noise area and the pixel point at the edge area of the segmented object, respectively. In this study, the simplest method of excluding the edge area from the density calculation was adopted. Thus, the threshold is calculated according to the number of points of the same color in the interval area, which is exactly the number of white points between the yellow and green lines. The left image can be regarded as the boundary case of point O1. If the value exceeds the threshold, the operation is set to 0. The threshold can be calculated using the following formula:


(8) $$\begin{array}{c} T\,h\,r\,e\,s\,h\,o\,l\,d=i^{2}-{\left(C\,e\,i\,l\,\left(\frac{i}{2}\right)\right)}^{2} \end{array}$$

where Ceil is a round-down operation. A mask is introduced for auxiliary operations:


M⁢a⁢s⁢k⁢B⁢l⁢a⁢c⁢k⁢(C⁢o⁢u⁢n⁢t⁢G⁢a⁢p⁢W⁢h⁢i⁢t⁢e⁢(X))

(9) $$\;=\left\{ \begin{array}{cc} 0,i\,f\,C\,o\,u\,n\,t\,G\,a\,p\,W\,h\,i\,t\,e\,\left(X\right)>T\,h\,r\,e\,s\,h\,o\,l\,d\; & \\ 1,o\,t\,h\,e\,r\,w\,i\,s\,e\; & \end{array}\right.,$$

M⁢a⁢s⁢k⁢B⁢l⁢a⁢c⁢k⁢(C⁢o⁢u⁢n⁢t⁢G⁢a⁢p⁢B⁢l⁢a⁢c⁢k⁢(X))

(10) $$\;=\left\{ \begin{array}{cc} 0,i\,f\,C\,o\,u\,n\,t\,G\,a\,p\,B\,l\,a\,c\,k\,\left(X\right)>T\,h\,r\,e\,s\,h\,o\,l\,d\; & \\ 1,o\,t\,h\,e\,r\,w\,i\,s\,e\; & \end{array}\right.,$$

The density matrix can then be expressed as


(11) $$\begin{array}{c} \begin{array}{c} D\,e\,n\,s\,i\,t\,y\,M\,a\,p\,X=R\,o\,u\,n\,d\,\left(X\right)\,\text{⋅}\,D\,e\,n\,s\,i\,t\,y\,W\,h\,i\,t\,e\,\left(X\right)\,\text{⋅}\,M\,a\,s\,k\,W\,h\,i\,t\,e\,\left(X\right)\\ +\left(1-R\,o\,u\,n\,d\,\left(X\right)\,\text{⋅}\,D\,e\,n\,s\,i\,t\,y\,B\,l\,a\,c\,k\,\left(X\right)\right)\,\text{⋅}\,M\,a\,s\,k\,B\,l\,a\,c\,k\,\left(X\right), \end{array} \end{array}$$

where Round indicates a rounding operation that acts as a shield. Generally, the lower the density of the point, the more likely it is to be within an outlier region, the greater the likelihood of misclassification, and the greater the need to assign a greater loss value. At present, DensityMap values are only calculated from the point density of the predicted results. Thus, during design of the proposed model, it was also necessary to integrate the BCE loss function.

Thus, the final outlier-region loss function of the proposed model can be expressed as


(12) $$\begin{array}{c} L_{o\,u\,t\,l\,i\,e\,r}\,\left(X,Y\right)=L_{B\,C\,E}\,\left(X,Y\right)-D\,e\,n\,s\,i\,t\,y\,M\,a\,p\,X, \end{array}$$

where *Y* is the label value with dimensions of *n* × *n*, the output is the loss value matrix with dimensions of *n* × *n*, and a negative sign is added in front of *DensityMapX* as the point density is inversely related to the loss value. The final loss value can be obtained using a sum or a mean function.

The outlier-region loss function is calculated based on essentially stable prediction results and assigns higher loss values upon detection of the small number of anomalous outlier regions. Thus, this function is more suited for use in combination with the conventional loss function in a weighted manner, with the conventional loss function dominating in the early stage of training. and the weights of the outlier-region loss function gradually being increased in the later stage once basic stability has been achieved.

The weighted fusion of the above loss functions is


(13) $$\begin{array}{c} L\,\left(X,Y\right)=\alpha \cdot L_{B\,C\,E}\,\left(X,Y\right)+\beta \cdot L_{D\,i\,c\,e}\,\left(X,Y\right)+\gamma \cdot L_{o\,u\,t\,l\,i\,e\,r}\,\left(X,Y\right), \end{array}$$

whereα, β, and γ are weight coefficients.

## 4 Experiment results and analysis

### 4.1 Experiment settings

#### 4.1.1 Dataset

**FIGURE 6:**
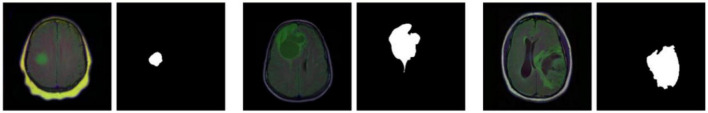
Partial images and fluid attenuated inversion recovery (FLAIR) segmentation results from the Lower-Grade Glioma (LGG) Segmentation Dataset.

The LGG Segmentation Dataset was used in this study, as obtained from https://www.kaggle.com/datasets/mateuszbuda/lgg-mri-segmentation. The number of MRI slices of patient brains ranges from 20 to 88, and the preoperative imaging data contain a fluid attenuated inversion recovery (FLAIR) sequence. This database includes approximately 3929 brain MRI images along with the corresponding manual FLAIR segmentation results, which are shown in Figure 6. Genomic data such as fDNA methylation, gene expression, DNA copy number, and microRNA expression and basic data such as age and gender are provided in a.csv file (Table 1). The values in the genomic data represent the molecular classifications of LGGs. For example, the RNASeqCluster column represents the molecular classifications of RNASeq, with values ranging from 1 to 4. For detailed explanations of each column, please refer to Buda et al. (1).

**TABLE 1:** Genomic data and basic data of some patients.

Patient	RNASeqCluster	MethylationCluster	miRNACluster	CNCluster	…	death01
TCGA_CS_4942	1	5	2	1	…	1
TCGA_CS_4943	1	5	2	1	…	0
…	…	…	…	…	…	…
TCGA_CS_5393	4	5	2	1	…	0
TCGA_CS_5396	3	3	2	3	…	0
TCGA_CS_6186	2	4	1	2	…	1

#### 4.1.2 Evaluation metrics

In this study, we used the DSC to evaluate the segmentation results. The DSC is an ensemble similarity measure function, which is usually used to calculate the similarity between two samples. With *X* and *Y* being the predicted and real results, respectively, the Dice coefficient is defined as


(14) $$\begin{array}{c} D\,i\,c\,e\,\left(X,Y\right)=\frac{2\,\left|X\cap Y\right|}{\left|X\right|+\left|Y\right|}. \end{array}$$

#### 4.1.3 Implementation details

This experiment was conducted on an Ubuntu server with an NVIDIA RTX 2060 graphics card. The segmentation_models.pytorch codebase was used, with some customization to incorporate the attribute encoder model. The training, validation, and test sets were scaled to 0.7, 0.15, and 0.15, respectively. In terms of data enhancement, the training set was processed by randomly deleting channels and applying random brightness, contrast, and saturation values. The Adam optimizer was used. The learning rate was set to 0.001 and the random number seed was set to 23.

In this experiment, a function was added to analyze the segmentation size in terms of the dataset. A total of six segmentation regions smaller than 7 × 7 were found in the dataset, with the proportion of such regions being less than $\frac{6}{3929}$. Thus, a segmentation area smaller than 7×7 in the segmentation result was considered to have a high probability of being segmented incorrectly and, in this experiment, the hyperparameters *i* and *j* were set to 7 and 21, respectively. For the outlier loss function, the hyperparameters α, β, and γ were all set to 1, and *i* and *j* could be freely set according to the size of the area to be excluded.

### 4.2 Ablation studies

#### 4.2.1 Loss function ablation

In this study, we used the BCE loss function as the baseline loss function and added other loss functions separately to compare the DSC and intersection over union (IoU) scores. The results are listed in Table 2.

**TABLE 2:** Ablation analysis of loss function.

Encoder	Decoder	BCE loss	Dice loss	Outlier loss	DSC	IoU
RegNet	DeepLabv3+	√			93.21	90.70
		√	√		93.86 (+0.65)	91.27 (+0.57)
		√	√	√	**94.36** (+1.15)	**91.83** (+1.13)

Bold values represents the best score.

**FIGURE 7:**
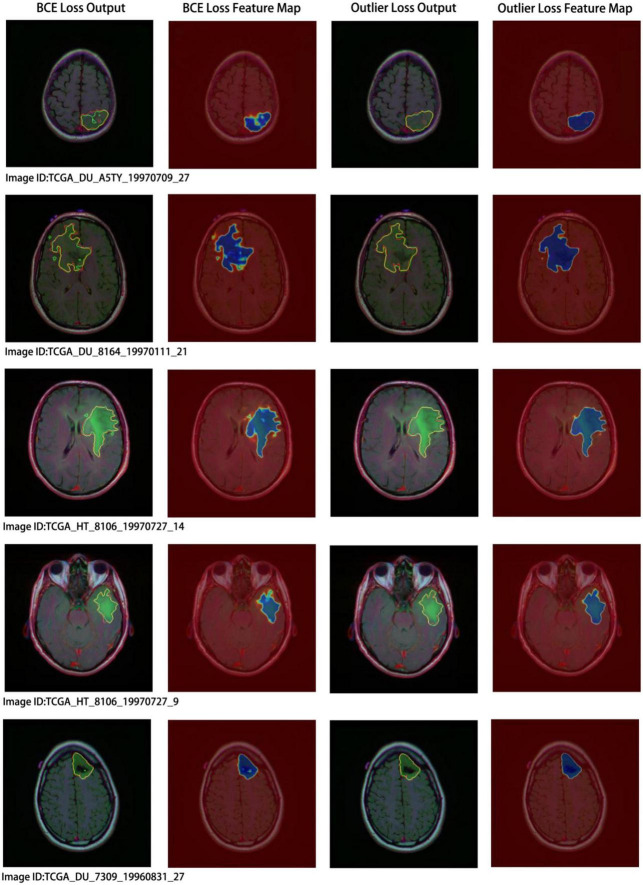
Segmentation results and visual analysis obtained using different loss functions. The first and second columns show the segmentation results and probability heatmaps obtained using the binary cross-entropy (BCE) loss, while the third and fourth columns show the segmentation results and probability heatmaps obtained using the outlier loss. In the segmentation results, the red line represents the ground truth label, and the green line represents the predicted result. The probability heatmap represents the confidence of the prediction: darker colors indicate higher confidence.

Concurrently, we performed a visual analysis on a subset of images, as shown in Figure 7. The first and second columns indicate the segmentation results and probability heatmaps obtained using BCE loss, whereas the third and fourth columns show the segmentation results and probability heatmaps obtained using outlier loss. The red and green lines in the segmentation results represent the ground truth and predicted labels, respectively. The probability heatmaps represent the confidence of the predictions: darker colors indicate higher confidence. From the comparison of the predicted segmentation results, it is evident that the use of outlier loss significantly reduces the occurrence of noise and improves the edge fitting effect, thereby greatly enhancing the quality of the segmentation results. Furthermore, in the comparison of the predicted probability results, the use of outlier loss leads to fewer intermediate colors in the edge regions, indicating higher confidence and better model performance. The foreground region exhibits a higher consistency in color, indicating a smaller variance in the overall predicted results for the foreground region and a more stable segmentation effect. In contrast, the use of BCE loss results in lower color consistency for the foreground region, indicating a larger variance in the overall predicted results for the foreground region and poor segmentation performance.

#### 4.2.2 Ablation analysis of multimodal data

This study conducted ablation experiments on models with and without attribute encoders to evaluate the impact of the attribute encoders on the results. The results are shown in Table 3.

**TABLE 3:** Ablation analysis of multimodal data.

Attribute encoder	DSC	IoU
No	92.80	90.22
Yes	**93.21**	**91.02**

Bold values represents the best score.

#### 4.2.3 Base model ablation

In this study, we performed a comparative analysis of several different groups of encoders using DeepLabv3+ as the main decoder; the results are presented in Table 4. The proposed RegNet model achieved the highest score.

**TABLE 4:** Ablation analysis of popular models.

Encoder	Decoder	DSC	IoU
U-Net (1)	U-Net (1)	82.00	–
ResNet (35) (resnet101)	DeepLabv3+	90.43	87.80
EfficientNet (36) (tf_efficientnet_b5)	DeepLabv3+	91.0	88.13
Xception (37) (xception65)	DeepLabv3+	91.65	89.10
RepVGG (38) (repvgg_b2)	DeepLabv3+	91.87	89.36
EfficientNetV (39) (tf_efficientnetv2_m)	DeepLabv3+	91.93	89.29
Swin Transformer v2 (40) (swin_base)	DeepLabv3+	91.98	89.19
RegNet (regnetz_040)	DeepLabv3+	**92.80**	**90.22**

Bold values represents the best score.

Notably, the recent transformer-based model (Swin Transformer v2) did not achieve acceptable results; this was because of the small dataset used in this experiment.

**FIGURE 8:**
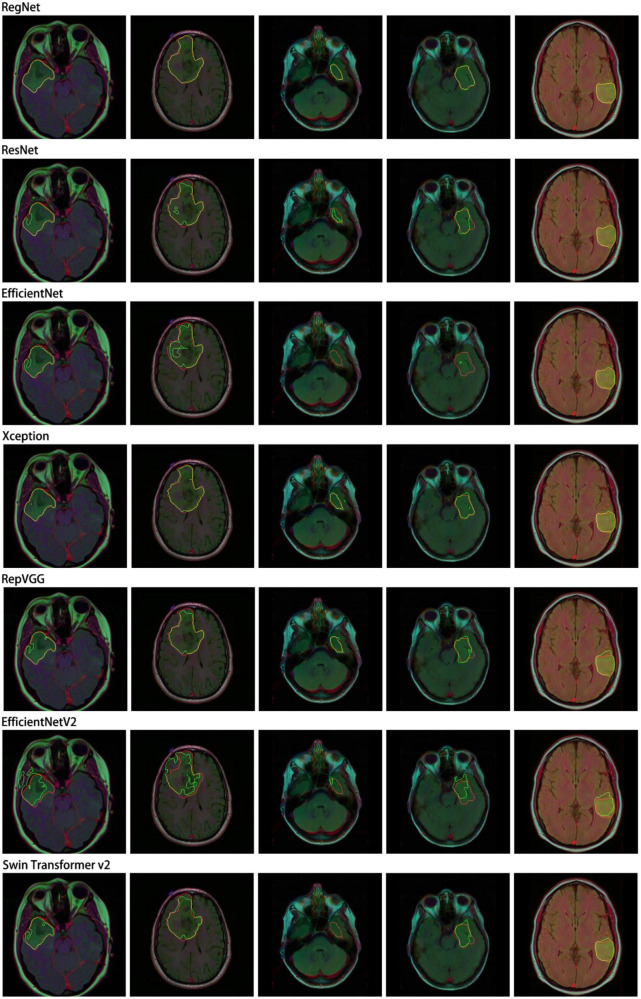
Segmentation results for different models. In the segmentation results, the red line represents the ground truth label, and the green line represents the predicted result. The image IDs are TCGA_FG_8189_20030516_21, TCGA_DU_8164_19970111_22, TCGA_CS_5397_20010315_7, TCGA_CS_5397_20010315_9, and TCGA_CS_6290_20000917_10.

Similarly, we selected some images for visual analysis, as shown in Figure 8, From the comparison of the segmentation results, it can be seen that using RegNet as the encoder of the model leads to better edge fitting and higher-quality segmentation results.

### 4.3 Outlier-region loss-function visualization

**FIGURE 9:**
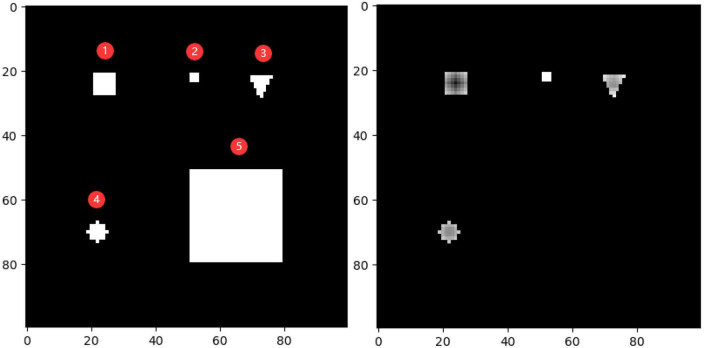
Test image (left) and loss value visualization (right).

To demonstrate the effectiveness of the outlier loss function more intuitively, a characteristic picture was manually designed and used as the outlier loss function input. The 100 × 100 input image and the loss value output are shown on the left and right of Figure 9, respectively. Here, different shades of gray represent different loss values, with a lighter shade indicating a higher value. The input image consisted of five types of white areas. Areas 1–4 had sizes of less than 7×7 and were used to simulate noisy regions with different characteristics. Area 5, which had a size of 28×28, was used to simulate a normal segmentation area. Hyperparameters *i* and *j* were set to 7 and 21, respectively. In the figure on the right, the noise regions in Areas 1–4 were given different loss values according to their density ratios compared to the background region. Area 1 was exactly within the size of the noise region; thus, it was darker overall and, correspondingly, had a lower loss value. The higher loss values of the edge parts were determined by the manner in which the density was calculated and did not affect the determination of the noise region. Area 2 had the highest loss value because it had the lowest density. Areas 3 and 4 simulated noisy regions with different shapes. Unlike Areas 1–4, Area 5 was not assigned a loss value because its overall size exceeded that of the noise region, and thus, it was judged to be a normal segmented region.

## 5 Conclusion

In this study, a deep learning-based model with an encoder–decoder structure was proposed for MRI image segmentation and application to glioma. The model obtained in the RegNet design space was applied as the encoder and combined with the decoder component of DeepLabv3+. In the experiment, excellent segmentation results were obtained. Additionally, to address the misclassifications occurring in outlier regions of a certain size, a density-based outlier-region loss function was proposed to suppress generation of such regions. Finally, multimodal data fusion in the LGG Segmentation Dataset was explored. Experiment results with a Dice score of 94.36 were obtained on the LGG Segmentation Dataset; thus, the proposed method had excellent performance. Moreover, this model outperformed other popular models.

As limitations, the application range and detection method of the proposed model can be improved. Therefore, in future research, the density-based outlier-region loss function will be extended for multi-class and three-dimensional segmentation, thereby improving the application range of this method. To explore superior detection methods, traditional outlier detection theory will be introduced. Moreover, the use of the Dice coefficient as the evaluation function is not intuitive as this method focuses on the noise removal efficiency. Thus, an evaluation function that can detect the number of noise points is more reasonable, and such a function will be studied in future research. Moreover, although the recently popular transformer-based vision model did not achieve the best results on the dataset considered in this work, it may perform better when its global modeling capability is combined with the advantages of the local extraction feature of traditional CNNs. Finally, further research is needed regarding quantification of the effects of data of other modalities on the final result and their influence scope.

## Data availability statement

The datasets presented in this study can be found in online repositories. The names of the repository/repositories and accession number(s) can be found below: https://github.com/intbingbing/LGG-Image-Segmentation.

## Author contributions

MZ and BW: conceptualization, methodology, and writing—review and editing. XY: software. KL and BW: validation. BH: writing—original draft preparation. All authors contributed to the article and approved the submitted version.
